# Hydrodynamic analysis of bioinspired vortical cross-step filtration by computational modelling

**DOI:** 10.1098/rsos.230315

**Published:** 2023-05-10

**Authors:** S. Van Wassenbergh, S. L. Sanderson

**Affiliations:** ^1^ Laboratory of Functional Morphology, Department of Biology, University of Antwerp, Universiteitsplein 1, 2610 Antwerpen, Belgium; ^2^ Department of Biology, William & Mary, 540 Landrum Drive, Williamsburg, VA 23187-8795, USA

**Keywords:** filter feeding, suspension feeding, crossflow filtration, porous media model, computational fluid dynamics, fish feeding

## Abstract

Research on the suspension-feeding apparatus of fishes has led recently to the identification of novel filtration mechanisms involving vortices. Structures inside fish mouths form a series of ‘backward-facing steps' by protruding medially into the mouth cavity. In paddlefish and basking shark mouths, porous gill rakers lie inside ‘slots’ between the protruding branchial arches. Vortical flows inside the slots of physical models have been shown to be important for the filtration process, but the complex flow patterns have not been visualised fully. Here we resolve the three-dimensional hydrodynamics by computational fluid dynamics simulation of a simplified mouth cavity including realistic flow dynamics at the porous layer. We developed and validated a modelling protocol in ANSYS Fluent software that combines a porous media model and permeability direction vector mapping. We found that vortex shape and confinement to the medial side of the gill rakers result from flow resistance by the porous gill raker surfaces. Anteriorly directed vortical flow shears the porous layer in the centre of slots. Flow patterns also indicate that slot entrances should remain unblocked, except for the posterior-most slot. This new modelling approach will enable future design exploration of fish-inspired filters.

## 1. Introduction

Suspension-feeding fishes such as tilapia, carp, anchovies and menhaden filter enormous volumes of water to extract and concentrate very small food particles. They belong to at least 21 diverse fish families in 12 orders, compose about 25% of the world fish harvest, and are critical links in the food webs of many aquatic ecosystems [1–3]. By contrast with the high ecological and economical importance of this group, we still know relatively little on how these fish manage to filter small, suspended food particles. Previous assumptions that the oral structures of fishes serve as simple mechanical sieves that act like a colander or coffee filter have not been supported [3,4]. Suspension-feeding fishes have been discovered to use crossflow filtration in which the mainstream flow of water (i.e. the feed flow) through the mouth cavity is directed tangential to (i.e. parallel to or across) filtering structures rather than perpendicular to the filter [5–7]. Only close to the porous surfaces of a crossflow filter, water flows bend away from the mainstream crossflow direction to pass through the porous filter surfaces. This new perspective has stimulated recent research on the complex fluid dynamics of water filtration inside the mouth cavity of suspension-feeding fishes [8–11] and has led to studies on the vortical flow patterns generated by fish filters during a unique crossflow process termed vortical cross-step filtration [9].

**Figure 1.**
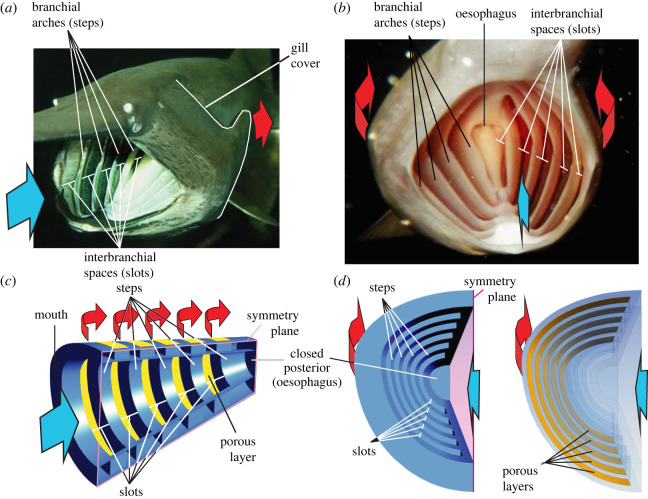
Morphology of cross-step filters. Head of a paddlefish (*Polyodon spathula*) during ram suspension-feeding in oblique frontolateral view (*a*) and in frontal view (*b*) showing the branchial apparatus consisting of steps (i.e. the branchial arches) separated by slots (i.e. interbranchial spaces within which the gill rakers are located). (*c,d*) A geometrically simplified model similarly consisting of steps, slots and a porous layer (in yellow) to represent the gill rakers. The models are shown in views matching the fish images above. In (*d*) the model is shown with non-transparent faces on the left, and with transparent faces on the right to show the porous layer located deep inside the slots. Blue arrows indicate water flow direction into the mouth, and the red arrows indicate final (opercular) outflow after the water has passed into the interbranchial slots and through the gill rakers and gills. A gill cover is not essential for cross-step filtration and is not used in the model. The geometry of the models is further illustrated in the electronic supplementary material, video S1 [14].

Unlike industrial filtration systems, fish filters consist of obstacles (e.g. branchial arches, gill rakers and denticles) that generate vortices owing to flow separation, just as bridge pilings and tree trunks generate eddies that swirl downstream [8,9]. Research on three-dimensional-printed models of fish filtering structures has shown that these vortices can suspend, concentrate and transport particles during vortical cross-step filtration [12,13]. In paddlefish (*Polyodon spathula*) and basking sharks (*Cetorhinus maximus*), the steps are formed by the sequentially arranged branchial arches that are directly exposed to the mainstream flow when these species ram suspension-feed by swimming forwards with an open mouth (figure 1*a,b*). Branchial arches are a series of separate bones between which water must flow to exit posteriorly from the fish mouth cavity during feeding and ventilation. Between neighbouring arches, a layer of comb-like gill rakers attaches to the arches and covers the bottom of each interbranchial slot (figure 1*a*,*b*). Owing to the conical shape of the mouth cavity, the radii of arches become smaller towards the narrower back of the mouth cavity (figure 1*b*). Although the oesophagus, situated at the very back of the mouth cavity (figure 1*b*), can swallow food and water by peristaltic action, oesophageal ingestion of water is negligible compared to the large volumes of water passing through the mouth cavity during the filtration process. Hence, from a fluid-dynamics perspective the oesophagus was treated as closed during this phase.

**Figure 2.**
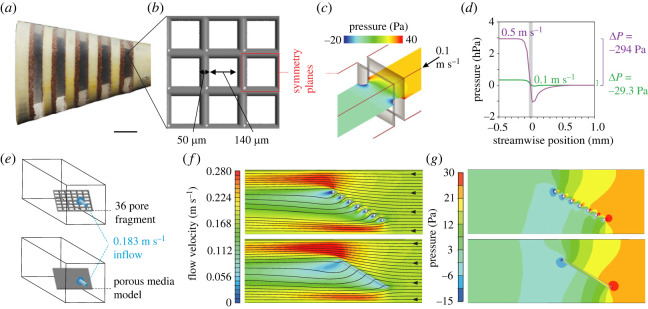
Characterization and validation of a porous media model for flow through a square-pored mesh. (*a*) Three-dimensional printed cross-step model covered on the exterior with a nylon mesh, showing particles concentrated primarily along anterior slot margins (modified after [9]). Scale bar, 1 cm. (*b*) Geometry of the nylon mesh with indication of a single pore with its symmetry planes in cross-section. (*c*) Output of computational fluid dynamics (CFD) simulations of perpendicular flow through the nylon mesh, showing the pressure drop across the filter surface at an approaching flow velocity of 0.1 m s^−1^. (*d*) Quantification of the pressure drop at different velocities, which is used to determine the input values for the porous media models. The grey bar shows the position of the pore. (*e*) Outline of a CFD validation test for flow at the nylon mesh fragment at an angle of attack of 30°, used in (*f*) and (*g*) for comparing output of a reference model with actual pores (top) to the output of a thin porous plate to which the porous media model is assigned in ANSYS Fluent (bottom). (*f*) Comparison of flow velocities and streamlines (black lines), and (*g*) pressures along the transverse section plane for the two CFD models shown in (*e*).

To be filtered, water travels over and between the branchial arches and into the slots, and then exits from the mouth cavity by passing through the pores between the gill rakers. While travelling over a branchial arch with a slot just downstream of it, the water will encounter what is known in the field of hydrodynamics as a ‘backward-facing step’, a well-known geometry for the development of flow separation and recirculation [15–17]. Particles are concentrated along the anterior slot margins (figure 2*a*). The vortical flows inside the slots have been revealed by dye visualization in preserved specimens of paddlefish in a flow tank [12] as well as in geometrically simplified physical models based on the anatomy of suspension-feeding fishes ([9]; figure 1*c*,*d*). In this paper, we build on the previous work involving physical models of fish-inspired cross-step filtration [13,18] to advance our knowledge and methodology on the hydrodynamics of cross-step filters, including the effects of altering porosity as a key variable.

The current lack of quantitative data on the three-dimensional dynamics of vortical water flows in fish-inspired cross-step filtration is hindering our mechanical insight. Computational fluid dynamics (CFD) can provide such data and can inform the design of morphological modifications for future models. CFD has become an indispensable research tool in various sciences (e.g. [19]), and has already proved very useful in the study of animal filtration systems (e.g. [5,11]). However, current types of models either do not include a porous layer [8], are limited to flow through a few pores [11], or have simplified porous layer geometries to flat plates [20,21]. The aims of the current study are to: (i) propose a modelling protocol that overcomes these limitations by outputting a full three-dimensional solution including flow through irregularly shaped porous surfaces; (ii) validate this CFD model against data available from flow tank measurements; (iii) use the model output to test the effects of modifying or eliminating the porous mesh used in the physical models, and supplement our current knowledge on flow patterns in a cross-step filter; and (iv) discuss the implications for cross-step filtration performance.

## 2. Material and methods

### 2.1. Computational fluid dynamics modelling strategy based on physical model structure

We developed and validated a CFD model of a fish-inspired cross-step filter used in previous experimental research: the physical model from Sanderson *et al*. [9] with the branchial arches at 90° (figure 1*c*,*d*). This physical model is covered on the exterior by a nylon mesh (figure 2*a*) with square pores of 140 µm edge length and a thread thickness of 50 µm (figure 2*b*). Since the number of pores spanning the branchial slots on one-half of the model exceeds 50 000 and CFD would require about 1 million grid elements for accurately solving flow through one pore (figure 2*c*), the computational costs would be excessive to include the actual pores of the nylon mesh. For this reason, methods to approximate flow though porous structures, without explicitly modelling the pores, are included in CFD software, namely porous media models. With these models, the resistance to water flow in three orthogonal directions needs to be defined. Firstly, we derived the input variables for this model from independent sets of CFD simulations of steady water flow though thin porous plates. Secondly, the direction of the main permeability (i.e. perpendicular to the nylon mesh surface of the physical model) needed to be set for each cell of the porous region (also referred to as the porous zone) where water exits from the CFD model of the cross-step filter. This is not trivial for irregularly shaped porous surfaces. Finally, flow though the cross-step model was solved.

As previous flow tank experiments showed that the major vorticity patterns are stable [9], and as each individual ram filter-feeding fish swims forwards at a roughly constant velocity (e.g. [22]), simulations of steady flow were appropriate and the most practical approach. Compared to alternative approaches using time-resolved CFD, this approach considerably decreases the computational cost, and simplifies the comparison among different models. However, it should be kept in mind that small-scale turbulence and potential temporal fluctuations in the intensities or location of the large vortices will not be resolved.

### 2.2. Porous media model

The porous media model of ANSYS Fluent (ANSYS 2019 R1; ANSYS Inc., Canonsburg, PA, USA) was used. This model adds a momentum sink in the governing momentum equations based on estimates of flow resistance in the porous region. The resistance is quantified as a pressure drop Δ*p* across the porous medium, and is assumed to be a combination of Darcy's Law and an additional inertial loss term:

2.1 $$\Delta p=\left(\;\frac{\mu }{\alpha }v+\;C_{2}\;\frac{1}{2}\rho v^{2}\right)\Delta m,$$

where *µ* is the dynamic fluid viscosity (µ = 0.00103 Pa s), *α* is the permeability of the medium, *C*_2_ is the pressure-jump coefficient, *v* is the velocity normal to the porous face, *ρ* is the fluid density (*ρ* = 998.2 kg m^−3^) and Δ*m* is the thickness of the medium (Δ*m* = 50 10^−6^ m). The two unknowns, *α* and *C*_2_, can be derived from a minimum of two CFD simulations with a different *v* input and resulting Δ*p* output (figure 2*d*) by substitution in equation (2.1). These simulations make use of symmetry boundary conditions at the four sides of each square pore (figure 2*b*,*c*). The pore walls were placed at 2 mm downstream of a velocity inlet. A pressure outlet (arbitrarily set to zero gauge pressure) was placed at 8 mm behind the pore, which is more than sufficiently far to resolve the small wake. Grid edge lengths were 1 µm at the pore faces, and were allowed to grow by 5% per layer away from the pore to a maximum of 1 mm edge length. This resulted in a mesh of 5.36 10^6^ cells. As a considerably coarser mesh of 1.48 10^6^ cells showed only 3% difference in pressure drop, the grid was considered sufficiently fine. Simulations at 0.1 m s^−1^ and 0.5 m s^−1^ gave, respectively, Δ*p* = −29.3 Pa and −293.6 Pa (figure 2*d*), and subsequently through equation (2.1), face permeability *α* = 2.3468 10^−10^ m^2^ and pressure-jump coefficient *C*_2_ = 29 473 m^−1^. As an additional validation, feeding these values back into equation (2.1) for a flow velocity of 0.183 m s^−1^ (the mainstream velocity of the flow tank experiments by Sanderson *et al*. [9]) predicts a pressure jump of −64.8 Pa, which corresponds well with the value of −64.0 calculated from the CFD using the actual square pore.

The above procedure calculates the resistance to flow perpendicular to the porous layer, but input values for resistance to other incident directions can still be fine-tuned. To do so, and to further validate the porous media model, simulations were performed of a porous plate fragment consisting of 36 actual pores at an angle of attack of 30° (figure 2*e–g*; top row) for comparison to the porous media model in the same configuration (figure 2*e*–*g*; bottom row). The flow domain and grid size were identical to the single pore simulations (see above), and the input velocity was 0.183 m s^−1^. As recommended in the Fluent User Guide (version 2019R1), the pressure-jump coefficient was set equal in all directions, but the face permeability was set lower tangential to the porous layer. Starting with a roughly 100 times lower value for tangential permeability with respect to the perpendicular permeability *α*, the pressure drop at 30° incident flow was 17% overestimated by the porous media model. After adjusting tangential *α* to 10^−10^ m^2^, flow velocity patterns and pressures closely matched (figure 2*f*,*g*).

### 2.3. Boundary conditions and permeability direction vector mapping

**Figure 3.**
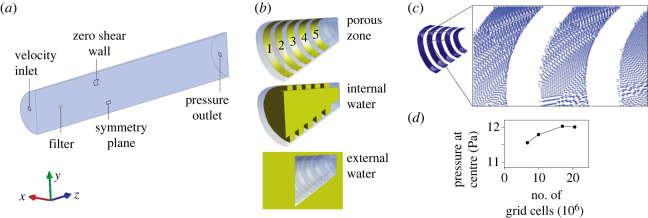
CFD boundary conditions, permeability direction mapping, and grid convergence. (*a*) Flow domain and boundary conditions of the exterior faces. The coordinate system axes orientation is illustrated on the bottom left corner. (*b*) Detailed view of the filter, oriented as in (*a*) with an open mouth, five porous slots (numbered 1 to 5 from anterior to posterior), and closed at the back. Three volumes (in green) are defined as separate entities: a ‘porous zone’ consisting of five thin layers of 50 µm thickness (one for each slot) to which the porous media model is applied, and the ‘internal water’ and ‘external water’ as standard fluid zones that are connected to each other at the mouth and to both sides of the porous zone. (*c*) Illustration of the vectors (arrowheads) defining the main direction of pore permeability for each cell of the porous zone. (*d*) Grid convergence analysis by monitoring pressure at the centre of the filter (inlet flow velocity = 0.183 m s^−1^), indicating convergence for the grid of 20.5 million cells.

The flow domain was set up as a 5 m long half cylinder (radius 0.4 m) with the longitudinal plane sectioning the modelled fish head midsagittally at 0.6 m from the inlet (flow speed = 0.183 m s^−1^) (figure 3*a*). The curved side face was modelled as a non-permeable surface allowing fluid to freely slip without shear force. The downstream base plane was defined as a pressure outlet set to zero gauge pressure. The no-slip (i.e. ‘wall’) condition was enforced at the non-porous surfaces of the filter. The length of the filter, from mouth centre to the posterior internal wall at the back, was 60 mm. The posterior wall of the cone is solid to simulate the closed oesophagus of the fish, but could be modelled as a porous surface or a specific outlet boundary condition if known.

The domain's fluid volume was divided into three entities as illustrated in figure 3*b*. The main fluid zones (‘internal water’ and ‘external water’) border the thin ‘porous zone’ volume. The physical properties assigned to the main fluid zones were those of normal water at 20°C (density 998.2 kg m^−3^, viscosity 1.003 mPa s). The two separate fluid zones connected to the porous zone are required for the following approach to define the permeability directions in each cell of the porous zone for an irregular surface. The porous zones are considered ‘irregular’ as these are parts of elliptical truncated cones, and hence no simple equation describes the direction of the surface normals.

The following steps were performed to map the permeability direction vectors on the porous zone cells. Firstly, ANSYS Fluent is used to calculate a gradient of a user-defined scalar (UDS) that has this direction. To do so, fixed values of UDSs are assigned for the separate fluid zones so that the downstream fluid zone, the ‘external water’ has a higher UDS value than the upstream zone, the ‘internal water’. Next, the unknown UDS values for the porous zone cells are solved numerically by Fluent in 25 iterations. After this, a user-defined function is executed to calculate the direction vectors (from the UDS gradient) and store them in user-defined memory (figure 3*c*). Finally, the direction vectors stored in memory were selected in the panel of the porous zone, and used in the flow calculations.

### 2.4. Solving cross-step computational fluid dynamics models

Steady flow simulations were performed by numerically solving the Reynolds-averaged Navier–Stokes equations. The flow domain originally consisted of approximately 20.5 million mesh cells (porous zone 65533; exterior water 10.7 10^6^; interior water 9.8 10^6^) constructed in ANSYS Meshing. The minimum edge length of the mesh to apply to regions of the highest interest, the internal surfaces of the filter, was set at 0.16 mm. Mesh element size was allowed to increase away from the filter at a growth rate of 1.08. The 50 µm thin porous zone was meshed with a single layer of hexahedral and wedge elements using the sweep method.

To account for the effects of turbulence associated with internal flows at Reynolds numbers (Re) above 4000 or in the transitional flow regime between 2000 and 4000 [23] (here Re at the mouth aperture = 4420 for a 40 mm hydraulic diameter, and 0.111 m s^−1^ posterior water flow), the four-equation shear stress transport (SST) model was used in the CFD solver ANSYS Fluent. This model combines two commonly applied models (the k-ε model in the free stream and the *k*-*ω* model near the walls), and has been shown to provide stable and close estimations of turbulence intensity in pipe flows [24]. This turbulence model, and the settings listed below, were also used in the abovementioned simulation to determine the porous media parameters (figure 2). Grid convergence was confirmed by observing a reduced and negligible per cent of difference (0.2%) in pressure at the centre of the filter (30 mm posterior of the centre of the mouth aperture) when refining the grid from 17 million to the chosen grid of 20.5 million cells (figure 3*d*).

The default solver settings of ANSYS Fluent 2019 R1 were used (SIMPLE scheme; least-squares cell based gradient treatment; second order pressure discretization; second order upwind for momentum; first order upwind for turbulent kinetic energy, specific dissipation rate, intermittency and momentum thickness Re). Calculations were run for 2000 iterations on a computer with 36 processor cores. Iterative convergence was monitored for drag force on the filter, together with the scaled residuals of the governing equations.

To assess the influence of porous zone characteristics on the flow patterns, the output of the first model described above was compared with a second model for which the porous resistance was halved (i.e. viscous resistance of 2.13 10^9^ m^−2^ in the main direction and 5 10^9^ m^−2^ for the in-plane directions; inertial resistance set to 14 737 m^−1^), and a third model without porous media (i.e. no resistance). The latter model has normal water properties assigned to the layer used previously as the porous zone.

### 2.5. Flow tank experiments

Flow tank experiments were performed using physical models from three-dimensional printing (fine polyamide PA 2200, Shapeways) that were identical to the ones used in CFD. The same experimental set-up as described in Sanderson *et al*. [9] was used, with matching flow speed (0.183 ± 0.003 m s^−1^; mean ± s.d., *n* = 3 trials): the model was mounted in the centre of a recirculating flow tank (18 × 18 × 90 cm working area, 100 l total volume) using a sting attached to the closed downstream end of the model. To expand the existing data on physical models with a surrounding nylon mesh [9] for validating the computational models, experiments with physical models without a porous layer (i.e. no surrounding nylon mesh) were conducted. These new physical models had the same dimensions as the original model but the slots where water exits from the model were not covered with a nylon mesh and therefore lacked the resistance caused by the mesh.

## 3. Results

### 3.1. Computational fluid dynamics validation with flow tank data

**Figure 4.**
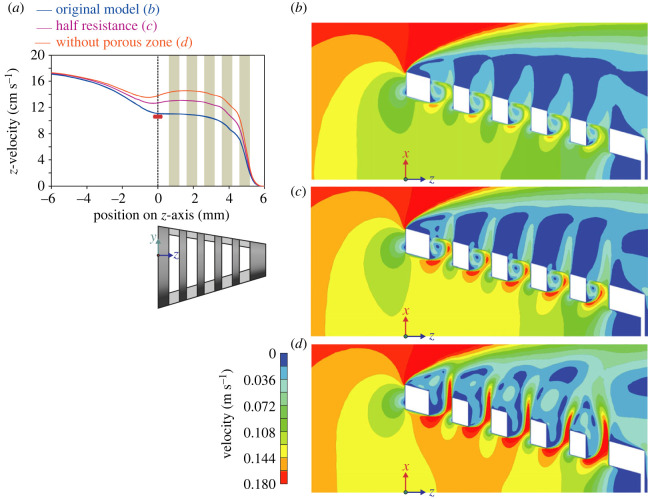
Velocity patterns in fish-inspired cross-step models with varying porous resistance. (*a*) Comparison of anterior-posterior flow velocity on the centreline (*z*-axis) of three CFD models, the original model with the resistance of the porous zone in the slots fine tuned as in figure 2 (in blue; corresponding to panel (*b*)), a model with half the porous resistance (in pink; corresponding to panel (*c*)), and a model without porous media and therefore without porous resistance in the slots (in orange; corresponding to panel (*d*)). The red box (± s.e.m.) and whiskers (± s.d.) show the velocity measured in a flow tank for physical models (*n* = 5) corresponding to the ‘original model’ [9]. Green bars indicate the position of the slots through which water exits from the model. (*b*–*d*) Velocity patterns (three-dimensional magnitude) along the *xz*-plane (i.e. frontal section) are shown as colour contour maps for the three models.

**Figure 5.**
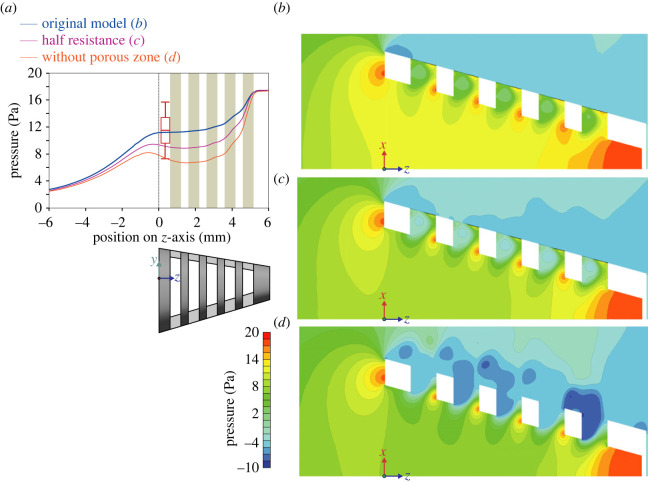
Pressure patterns in fish-inspired cross-step models with varying porous resistance. (*a*) Comparison of pressure on the centreline (*z*-axis) of three CFD models, the original model with the resistance of the porous zone in the slots finetuned as in figure 2 (in blue; corresponding to panel (*b*)), a model with half the porous resistance (in pink; corresponding to panel (*c*)), and a model without porous resistance in the slots (in orange; corresponding to panel (*d*)). The red box (± s.e.m.) and whiskers (± s.d.) show the pressure measured in a flow tank for physical models (*n* = 5) corresponding to the ‘original model’ [9]. Green bars indicate the position of the slots. (*b*–*d*) Pressure patterns (three-dimensional magnitude) along the *xz*-plane (i.e. frontal section) are shown as colour contour maps for the three models.

**Figure 6.**
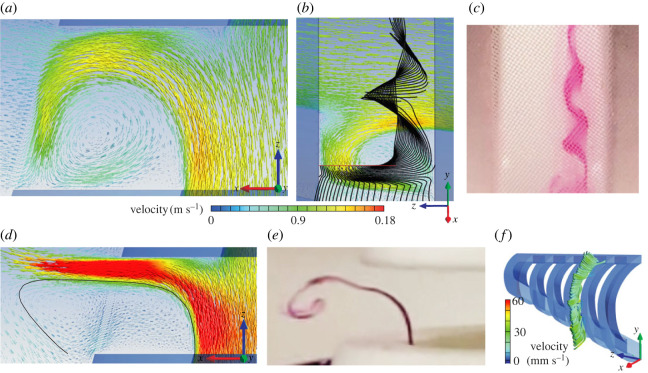
Flow patterns at the second slot and comparison with dye visualisations. (*a–c*) Data from the original model with a porous zone (corresponding to figures 4*b* and 5*b*). Flow velocity vectors at the midfrontal section plane from CFD are coloured and scaled by velocity magnitude in (*a*) a dorsal-to-ventral view and (*b*) an inclined lateral view. In (*b*), black lines are streamlines released from equally spaced points on the red line. (*c*) Helical vortex from dye visualisation in a flow tank (from [9]). (*d–f*) Data for the model without a porous zone (i.e. zero porous resistance), showing (*d*) flow velocity vectors from CFD and (*e*) dye released from the edge of the rib just upstream of the second slot corresponding to the black streamline in (*d*). (*f*) Vortex streamlines coloured by velocity magnitude showing that the absence of the mesh causes the position of the vortex core to shift outside the filter.

**Figure 7.**
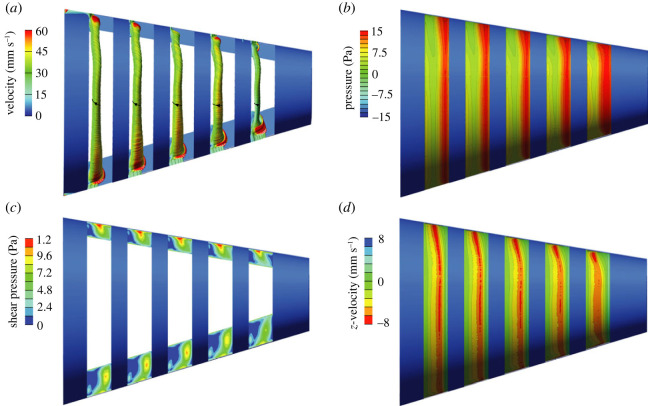
Intra-slot hydrodynamics in the cross-step filter. Results are shown for the original model (corresponding to figures 4*b*, 5*b* and 6*a*,*b*), viewed from the exterior of the model. (*a*) Streamline tubes coloured by velocity magnitude showing a helical vortex inside the anterior half of each slot. Black arrows show flow direction of the helix. (*b*) Pressure on the porous layer showing highest pressure at the posterior side of each slot where most flow exits (figure 4*b*), and a close-to-zero pressure in the centre. (*c*) Shear pressure at the medial edge surfaces of the slots showing central spots of high shear. (*d*) Anterior-to-posterior flow velocity magnitudes showing strong shear flows at the porous zone in the inverse direction (i.e. posterior-to-anterior) at the centre along the length of each slot.

The porous media CFD simulation (original model) output was compared with matching data from flow tank experiments using thermistor flow-velocity probes, pressure sensors and dye visualization [9]. CFD calculated a velocity of 0.111 m s^−1^ (figure 4*a*,*b*) and a pressure of 11.2 Pa (figure 5*a,b*) at the centre of the mouth aperture. The corresponding empirical measurements gave 0.105 ± 0.003 m s^−1^ and 11.5 ± 4.2 Pa (red boxes in figures 4*a* and 5*a*). Both in CFD and in the flow tank, a helical vortex was present at the anterior two-thirds of each slot in the original model (figures 4*b*, 6*a*–*c* and 7*b*). Outward flow speed in the posterior third of slot 2 at the midfrontal level was 0.105 ± 0.003 m s^−1^ in CFD (standard deviations for values at equally spaced points on a line segment), which corresponds well with the experimental measurement of 0.097 ± 0.006 m s^−1^ [9]. Velocity along the axis of the helix of the vortices varied among the slots between 0.022 and 0.0275 m s^−1^ at the midfrontal plane (y-velocity in the centre of the vortex) in CFD, while the experimental measurement was 0.030 ± 0.003 m s^−1^. The zone in which particles were cleared from the covering nylon mesh in the original physical model (figure 2*a*) corresponds well with the zone of near zero pressure (figure 7*b*) and strong shear flows (figure 7*c*,*d*) in the CFD model.

The CFD simulation without a porous zone (i.e. lacking a porous media resistance in the slots) showed flow patterns matching novel results from flow tank experiments using physical models that lacked nylon mesh. Helical vortices formed outside the filter (figure 6*d*). Axial velocities within these exterior helical vortices varied locally (see also figure 6*f*), and were of comparable magnitude in CFD (between 0.014 and 0.028 m s^−1^) and in the physical model (0.014 ± 0.006 m s^−1^). Linear speeds at the outer streamlines of the helical vortex were about 0.03 m s^−1^ on the anterior side (figure 6*f*), and up to 0.05 m s^−1^ on the posterior side in CFD. In the flow tank, these linear speeds were 0.0356 ± 0.0016 m s^−1^.

### 3.2. Flow patterns

When comparing consecutive slots 1–5 (figure 3*b*) in the CFD simulations, flow patterns near and inside of these slots showed a similar pattern (figure 4*b*–*d*), despite the narrowing of the conical filter and changing flow near the medial axis. At this medial axis of the filter, anterior-to-posterior flow velocities (along the *z*-axis of the model) were approximately constant at the level of the anterior two slots (1,2), and reduced slightly at the level of the next two slots (3,4) by, respectively 4% and 16% relative to the entrance velocity (figure 4*a*). At the level of the fifth (posterior-most) slot, *z*-velocity dropped off quickly (figure 4*a*). Pressure essentially followed the inverse pattern while increasing towards the closed back of the filter (figure 5*a*).

For simulations with a non-zero porous resistance in the slots (i.e. the original model and the half resistance model), outflow was present across the entire porous zone (figure 6*a*), but strongest at the posterior third of each slot where the water exerted a high pressure on the porous layer (figure 7*b*). At the middle third of each porous zone, flow became approximately parallel with the porous zone and anteriorly directed (figure 6*a*; electronic supplementary material, video S1 [14]). Flow velocity decreased towards the anterior third of the porous zone as filtrate continuously exited the stream shearing the porous zone. In the physical models of Sanderson *et al*. [9], small particles were concentrated on the mesh primarily along the anterior margin of the slots (figure 2*a*), to be transported to the oesophagus for swallowing at a later stage that has not yet been included as a component in physical or computational models of fish feeding. Sanderson *et al*. [9] and Schroeder *et al*. [13] have demonstrated that the vortices in physical models can be manipulated to transport small suspended particles downstream inside the slots. A large part of the slots was filled with a helical vortex (figures 6*a* and 7*a*). The direction of flow in the helix was from the side with the larger angle of attack, the ventral side of the model, to the less inclined side, the dorsal side (figure 7*a*). Along streamlines of the helical vortex, in general, flow accelerated at the posterior half and decelerated at the anterior half.

### 3.3. Effects of porous media parameters

Halving the resistance in the porous zone of the original model resulted in an increase in velocity of the entering flow at the centre of the mouth aperture by 14% (figure 4*a*), while pressure reduced by 17% (figure 5*a*). The peak flow velocity inside slot 2 increased by 20%.

Completely removing the porous media resistance resulted in a velocity increase by 23% at the centre of the mouth (figure 4*a*) and a pressure reduction of 30% (figure 5*a*). In the absence of porous resistance at the slots, the vorticity pattern changed notably (figures 4*d* and 6*d*–*f*). The vortex cores formed about 2 mm outside the filter instead of forming inside the slots. The posterior third of each slot showed a high-velocity stream of water outflow, reaching speeds that were 43% higher than the model with the original porous resistance. The middle and anterior portion of each slot showed low-velocity water (about 0.015 m s^−1^) entering the slots from the exterior (figure 6*d*).

### 3.4. Particle blockage of slot entrances

**Figure 8.**
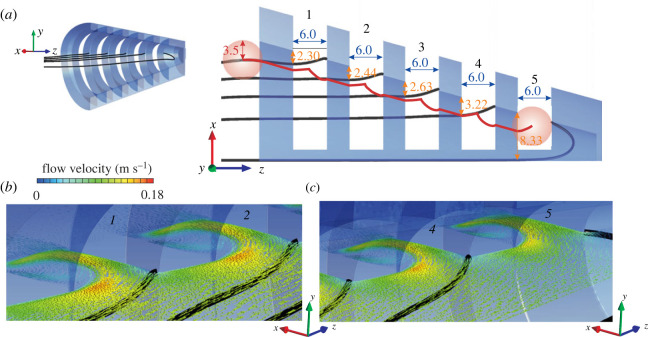
Flow directions and predicted path for large particles. Results are shown for the original model (corresponding to figures 4*b*, 5*b*, 6*a*,*b* and 7). (*a*) Dividing streamlines (in black) for flow into the consecutive slots (labelled 1 to 5) along the midfrontal section plane of the cross-step filter (left: oblique posterior-lateral viewing direction; right: dorsoventral view). All distance values (arrows) are in mm. Orange numbers (top-down distance arrows) show the minimal distance of the dividing streamline to the filter's surface. The red line estimates the path of a 7 mm diameter particle, assuming no elastic rebound upon impact with the boundaries, and following the flow direction in the absence of the particle as a first approximation. (*b*) Detailed oblique view on the flow field at the entrance of slots 1 and 2 and (*c*) 4 and 5. Flow velocity vectors are scaled and coloured by velocity magnitude

To estimate the size of spherical particles that can potentially block the entrances to the interbranchial spaces, streamline patterns were analysed (figure 8). Dividing streamlines were identified at the separation between the streamline entering a certain slot and the streamline continuing medially. The distance from a dividing streamline entering a slot to the interior surface of the filter gives an estimate of the maximum radius of particles that can enter the slot. For the first three slots, this distance is smaller than half the spacing between the slot walls, indicating no potential for blockage of the entrance to the slot. For each slot, a trajectory prediction can be given for a spherical particle of 3.5 mm radius, following the flow direction in the absence of the particle as a first approximation (Stokes number = 0), and assuming no elastic rebound upon impact with the boundaries (figure 8*a*). For the fourth slot, a small chance of blocking exists, namely for particles between 3 and 3.22 mm radius. For the fifth slot, all the water in the centre of the filter is drawn into the slot (figure 8*c*), which theoretically may provide transport towards the slot entrance of all sizes of particles that still fit into the conical posterior end of the model.

## 4. Discussion and conclusion

### 4.1. Computational fluid dynamics strategy and validation

We have developed and applied a CFD approach using the porous media model and permeability direction vector mapping in ANSYS Fluent to resolve fluid flow through a bioinspired cross-step filter. This strategy allows simulation of three-dimensional fluid flow through irregularly shaped porous layers. Previously, to our knowledge, a porous media model has only been applied once to assess flow patterns in an abstraction of a suspension-feeding animal [21]. In that study, fringes of the baleen of whales were modelled as a porous medium in a simplified, flat-plate configuration. Our new approach now eliminates the need to formulate mathematical equations to predefine permeability direction vectors for each cell of the porous layer. Instead, it makes use of the CFD software's finite-volume solver capacity to compute the shortest path across a porous layer, and maps the calculated direction vectors to the porous media model.

This new approach enables modelling of filters with porous layers that have shapes beyond simple planar or radially symmetric shapes. This is a crucial advancement, as among the highly diverse and abundant suspension-feeding taxa of metazoans [25], planar shapes or radial symmetry along the body axis is relatively rare. In addition to potential application to any three-dimensional scan-based geometry, the computational efficiency of porous media models makes it possible to perform CFD of entire mouth cavities of fishes on a standard workstation computer. Consequently, application in future studies of filtration mechanisms in vertebrate and invertebrate suspension feeders can be expected, and can guide the design of novel commercial and industrial filters.

This approach has been validated by comparing our simulation results and empirical experiments on physical models of cross-step filtration inspired by the mouth cavities of suspension-feeding fishes. The close congruence between the output of the CFD model and the measurements from physical models in a recirculating flow tank demonstrates that the details of complex flow structures can be resolved using this strategy. Optical techniques such as laser Doppler velocimetry cannot be used in the current physical cross-step models because the covering of nylon mesh obscures the view inside the slots. Therefore, this CFD model is ideal for quantifying three-dimensional parameters such as flow velocity gradients, pressure gradients, and shear pressures that provide crucial information on the functioning of cross-step filters.

### 4.2. Hydrodynamics of cross-step filtration

The hydrodynamics of vortical flow in the slots of a cross-step filter are non-intuitive and have been described only recently [12,18]. Our CFD model expands the scope of previous research by computing the locations of streamlines in three dimensions that visualize the direction and velocity magnitude of vortical flow within the slots (e.g. figure 7*a*). Streamlines can also illustrate the paths of fluid travelling within the vortices versus exiting from the slots (figure 6*b*). A novel result presented by this manuscript is that the porous zone in the CFD model is essential for generation of the flow profile responsible for cross-step filtration in the current physical models. This result is non-intuitive because at this Reynolds number all obstacles generate recirculation regions owing to flow separation [17]. Our results indicate that a porous resistance along the exterior of the slots in the physical model (figure 1*c*,*d*) is responsible for confining the vortices within the slot. In the absence of the porous layer, the vortex cores formed externally of both the CFD model and the physical model rather than remaining within the slots (figures 4*d* and 6*d*–*f*). This unexpected result could stimulate future research on variable porosity and the factors responsible for retaining the vortex within the slot.

Empirical measurements for calculating the porous resistance of the branchial apparatus during ram suspension-feeding are not available for live fishes. Fishes can change the porosity of branchial structures by abducting and adducting the branchial arches, and paddlefish and basking sharks can vary the porosity of the gill rakers across the slots between the branchial arches [26,27]. Werth [28] documented the ubiquity of variable rather than fixed porosity in biological filters. The CFD approach presented here will enable testing of hypotheses for prey retention across a range of gap sizes and porosities in multiple species.

Our results demonstrate that the generation of vortical flow structures in cross-step filtration is sustained over a range of values for porous resistance. When the resistance of the porous layer in the original CFD model was halved, pressures decreased inside the model (figure 5*b*,*c*) as flow velocities increased (figure 4*b*,*c*). The vortex cores became more pronounced inside the slots and the speed of flow exiting from the slots increased, but the basic vortical flow patterns persisted. Therefore, by modifying the porosity of the filter (branchial arches, gill rakers, denticles) during cross-step filtration, as well as by altering swimming speed, ram suspension-feeding fishes may be able to adjust vortical flow in and around the slots in response to particle properties such as size and shape.

### 4.3. Particle trajectories

We have focused on patterns of fluid flow during cross-step filtration, but this CFD model can also provide insight into particle movement. For example, the simulations can estimate the sizes of particles that will be excluded from the slots (figure 8). The distance between the interior filter surface and the dividing streamline that enters a given slot (orange numbers in figure 8*a*) indicates the theoretical maximum radius of a spherical non-deformable particle that can enter that slot. The trajectory for a particle with a radius larger than this maximum is predicted to be excluded from the slot entrance. This transport of particles towards the posterior of the fish mouth cavity has been observed endoscopically in live suspension-feeding goldfish, gizzard shad, and ngege tilapia as particles ‘slid’ or ‘bounced’ along the filter surfaces [5], and is similar to models of a ‘ricochet’ of particles away from manta ray filter surfaces on contact [11]. Although more accurate methods exist to determine particle trajectories by solving equations of motion of spheres [11], or by discrete-element modelling [21], analyses of dividing streamlines using the approach presented here can be informative as a first approximation of particle size selectivity that is accessible to a broader range of researchers. Further evaluations of this approach, for example by comparing its predictions with how cross-step filters handle particles of different sizes, would be valuable.

### 4.4. Future of computational fluid dynamics modelling strategy

Backward-facing steps are common structures in engineering applications and nature (review in [17]). In crossflow, a backward-facing step generates vortices downstream of the step (e.g. figures 4 and 5). When consecutive backward-facing steps are arranged relatively close together to form a series of *d*-type steps and slots (i.e. square grooves in a duct; aspect ratio of slot width to step height ≲3–4), each downstream vortex is isolated within the slot (e.g. [29,30]). Such *d*-type grooves are a common type of corrugated wall in ducts designed to reduce pressure losses and improve heat exchange (e.g. [30]). The design of cross-step filtration is unique compared to previous engineering applications of *d*-type backward-facing steps because the bottom of each slot between the steps of a cross-step filter is porous rather than solid. Water is allowed to exit from the porous regions of the slots between the steps of a cross-step filter, while the vortices continue to remain trapped within the slots [9,13].

Intraoral structures that are backward-facing steps with *d*-type configurations in crossflow are found in an enormous diversity of suspension-feeding vertebrates ranging in size from tadpoles to whales [9,31]. This diversity provides rich opportunities to explore cross-step filtration using CFD. For example, while a recirculation region has been reported to occur across Re ranging from 10^−4^ to 10^5^ and obstacle heights from 100 µm to the size of islands [32], CFD is the most practical approach for testing the operation of cross-step filtration across a biologically relevant range of flow velocities, pressure and shear forces, and porosities that correspond to differing size scales and therefore Re. In addition, the effects of varying biologically relevant structural features such as the angles of the slot walls and the number of slots can be quantified using these CFD methods. The process of cross-step filtration involves multiple morphological and hydrodynamic parameters that can be modified with the goals of understanding filter function and improving performance.

Measurement of the gap size or pore size between fish gill rakers is a standard method for assessing prey retention abilities in multiple orders (e.g. [33,34]). The CFD methods developed here for applying a porous media model to simulate flow patterns will be useful in a diversity of suspension-feeding fish species for which gap size has been compared to prey size, including species reported to use filtration mechanisms other than cross-step filtration (e.g. [35,36]). CFD offers a powerful strategy for testing the independent effects of controlled adjustments in oral structures and key fluid dynamic variables such as filter porosity and fluid velocity that have been reported to vary among suspension-feeding fish species as well as within species during ontogeny (e.g. [37–39]).

## Data Availability

All CFD data supporting the findings of this study have been made available in the Dryad Digital Repository [40]. The data are provided in the electronic supplementary material [14].
